# Parametric Electromagnetic Analysis of Radar-Based Advanced Driver Assistant Systems

**DOI:** 10.3390/s20195686

**Published:** 2020-10-05

**Authors:** Simona Vermiglio, Victor Champaney, Abel Sancarlos, Fatima Daim, Jean Claude Kedzia, Jean Louis Duval, Pedro Diez, Francisco Chinesta

**Affiliations:** 1 ESI Group, 3bis rue Saarinen, 94528 Rungis, France; ext-Simona.Vermiglio@esi-group.com (S.V.); victor.champaney@ensta-paris.fr (V.C.); Abel.SancarlosGonzalez@esi-group.com (A.S.); Fatima.Daim@esi-group.com (F.D.); Jean-Claude.Kedzia@esi-group.com (J.C.K.); Jean-Louis.Duval@esi-group.com (J.L.D.); 2 PIMM Lab & ESI Group Chair, Arts et Metiers Institute of Technology, 155 Boulevard de Hopital, 75013 Paris, France; 3 Laboratori de Calcul Numeric, Universitat Politecnica de Catalunya, Jordi Girona, E08034 Barcelona, Spain; pedro.diez@upc.edu; 4 Instituto Universitario de Ingenieria Mecanica y Biomecanica (I2MB), Universidad Politecnica de Valencia, Camino de Vera, s/n, 46022 Valencia, Spain

**Keywords:** computational electromagnetism, unwrapping, radar, ADAS, PGD

## Abstract

Efficient and optimal design of radar-based Advanced Driver Assistant Systems (ADAS) needs the evaluation of many different electromagnetic solutions for evaluating the impact of the radome on the electromagnetic wave propagation. Because of the very high frequency at which these devices operate, with the associated extremely small wavelength, very fine meshes are needed to accurately discretize the electromagnetic equations. Thus, the computational cost of each numerical solution for a given choice of the design or operation parameters, is high (CPU time consuming and needing significant computational resources) compromising the efficiency of standard optimization algorithms. In order to alleviate the just referred difficulties the present paper proposes an approach based on the use of reduced order modeling, in particular the construction of a parametric solution by employing a non-intrusive formulation of the Proper Generalized Decomposition, combined with a powerful phase-angle unwrapping strategy for accurately addressing the electric and magnetic fields interpolation, contributing to improve the design, the calibration and the operational use of those systems.

## 1. Introduction

Radar is a widely employed technology which relies on wave propagation to detect surrounding objects. It consists in emitting a radio wave from a transmitter and measuring the reflected wave with a receiving antenna. The data collected from this measurement can provide information about a detected object such as its location or its nature. An in-depth analysis of the electromagnetic field may be necessary to ensure the information inferred from the data is valid.

Radio waves are oscillations of the electromagnetic field and can therefore be computed by solving Maxwell’s equations. In the present work the electromagnetic problem is solved using the Finite Differences Time Domain (FDTD) method implemented in CEM One^®^, a commercial software provided by ESI Group, then computing the discrete Fourier transform of the result to obtain the solution in the frequency domain.

The fundamentals of radar technology is nowadays used in many driver assistance systems, needing for a precise quantification of performances and limitations [1]. Its design and use need addressing many topics, raging from waveform design, propagation and pattern recognition [2,3,4], with challenges and opportunities that are driving their evolution [5]. However, its use is not limited to driver assistance, this technology is nowadays largely employed in many domains like short-range localization [6], monitoring worker activity [7], aids for visually impaired people [8], physiological monitoring [9], non-contact identity authentication [10], among many others. Signal pollution is not excluded and sometimes it must be repaired before making use of it [11].

In outdoor applications, the transmitter and antenna are placed behind a radome, a shell made of a dielectric material designed to protect the electric components against the weather. Although dielectric materials are radio-wave transparent, they have an altering effect on the electromagnetic field and must therefore be taken into account when analyzing the data. Simulation of radio wave propagation through the radome provides knowledge of the latter’s precise influence on the system accuracy and performances. Thus, an important issue concerns the effect of bumpers on the radar performances, topic that attracted great interest [12,13,14,15,16,17]. In the just referred works the coupling radar-radome effects (e.g., attenuation, signal pollution, …) was investigated but a parametric numerical modeling was not considered.

In our work we address precisely the analysis of that coupling, depending on the radar orientation, however because of the high frequency, the spatial mesh resolution for describing the solution of Maxwell equations leads to extremely fine meshes, with the consequent impact on the computing time. Simulation details are included in Appendix B.

To alleviate that issue, we investigate the use of model order reduction (MOR) techniques that successfully accomplished numerous parametric studies in other engineering domains. Model order reduction has been successfully applied in problems involving dynamics and waves within the so-called projection formulation, needing for a certain degree of intrusiveness when using commercial softwares. Thus, the radial approximation proposed in [18] was extended to mid-frequency dynamics within the so-called variational theory of complex rays [19]. In [20], a parametric solution of the Helmholtz equation was successfully obtained using the usual rank-one greedy PGD constructor. In [21] authors proposed a consistent reduced bases interpolation.

Non-intrusive formulations were proposed for constructing parametric solutions from a number of high-fidelity simulations performed for different choices of the model parameters, while trying to reduce as much as possible the size of the sampling for addressing multi-parametric models [22,23]. In those circumstances, usual surrogate models exhibit limitations when the number of parameters increases, and alternative technologies combining two main ingredients, the separation of variables and sparse sampling and approximations, appeared and proved their performances [22,24].

When these techniques were employed in radar engineering different difficulties appeared. The first related to the fact that the sampling scales with the characteristic length of the solution, that is with the wavelength, extremely small. On the other hand interpolation of complex-valued electric and magnetic fields produces spurious solutions. Thus, for example, the average of 1+0i and −1+0i results 0+0i, even if one is expecting having 0+i.

This limitation was solved by employing and alternative formulation based on the amplitude and phase. For example, in the scenario just described, the average of 1|0 and 1|π results 1|π/2, result that seems more physically consistent.

However, the use of an amplitude/phase description faces the difficulty related to its 2π periodicity, and the associated spurious discontinuities found when combining a phase close to 2π, e.g., 2π−θ (θ>0, very small), with another very close too, e.g., $2\pi +\theta^{\prime }$ ($\theta^{\prime }>0$, very small), but that being higher than 2π reduces to $\theta^{\prime }$, originating the just referred spurious discontinuity: $2\pi -\theta \to \theta^{\prime }$. Even if this issue was addressed in many works that proposed the use of the so-called unwrapping, usual unwrapping algorithms only performs well when the data sampling is dense enough, but they fail in the sparse sampling case [25,26,27].

The present paper proposes a technique able to conciliate unwrapping and sparse sampling, technique that constitutes the most important scientific achievement of the preset work.

Before introducing the problem statement in Section 2, the proposed strategy in Section 3 and proving its performances in Section 4, we are revisiting the main key features of non-intrusive PGD-based model order reduction in the last part of the present introduction section.

### Model Order Reduction

A generic problem in physics consists of a differential operator L(·) acting on the so-called unknown field (here without loss of generality assumed scalar) u(x,t), with $x\in \Omega_{x}\subset R^{3}$ and $t\in \Omega_{t}\subset R$. The problem can involve a series of parameters grouped in vector μ ($\mu \in \Omega_{\mu }\subset R^{P}$) that being part of the optimization process, one would like to retain explicitly in the problem solution, i.e., u(x,t;μ).

The separated representations at the heart of the so-called proper generalized decomposition (PGD), initially proposed in the space-time settings [18] to generalize the proper orthogonal decomposition (POD) [28], were extended to represent parametric solutions of parametrized problems [29]

(1) $$u\left(x,t,\mu_{1},\cdots ,\mu_{P}\right)\approx \sum_{i=1}^{M}X_{i}\left(x\right)T_{i}\left(t\right)\prod_{j=1}^{P}M_{i}^{j}\left(\mu_{j}\right),$$

enablig simulation, optimization, inverse analysis, uncertainty propagation and simulation-based control, all them under real-time constraints [28,29].

However, using such a separated representation becomes very intrusive in the sense that specific algorithms are needed for sequentially compute the different functions involved in (1) by solving 3D partial differential equations (PDE) for computing functions $X_{i}\left(x\right)$, 1D ordinary differential equations (ODE) for calculating the time functions $T_{i}\left(t\right)$ and a series of algebraic problems for obtaining the functions depending on the parameters $M_{i}^{j}\left(\mu_{j}\right)$.

To reduce the intrusiveness, different strategies were proposed, among them the so-called Sparse Subspace Learning (SSL) [22] and its sparse counterpart, the so-called sPGD [24] revisited below.

If we consider the parameters $\mu_{1},\ldots ,\mu_{P}$ involved in the model grouped in vector $\mu \in \Omega_{\mu }$, the sampling constituting the DoE results in the parameters choices $\mu_{k}$, k=1,…,K, trying to cover as much as possible the parametric domain $\Omega_{\mu }$, to limit as much as possible extrapolation.

Now, from the computed solutions at $\mu_{k}$, noted by $u_{k}\left(x,t\right)$, one could try to infer the solution for any other value of the parameter μ by using a simple interpolation, as usually considered when constructing surrogate models,

(2) $$u\left(x,t;\mu \right)\approx \sum_{k}u_{k}\left(x,t\right)N_{k}\left(\mu \right).$$

However, being the parametric space in general high-dimensional standard interpolation, e.g., kriging, radial or polynomial approximation bases,…represented by the functions $N_{k}\left(\mu \right)$, fail for accomplishing an accurate enough approximation in the low-data limit, i.e., K≪P, limit in which even linear regressions do not work.

The sparse-PGD (sPGD) strategy proposed in [24] considers a separated approximation

(3) $$u\left(x,t;\mu \right)\approx \sum_{i}\prod_{n}U_{i}^{n}\left(x,t,\mu_{n}\right),$$

with

(4) $$U_{i}^{n}\left(x,t,\mu_{n}\right)=\sum_{j}w_{j}^{i,n}\left(x,t\right)F_{j}^{i}\left(\mu_{n}\right),$$

where standard polynomial bases are considered in $F_{j}^{i}\left(\mu_{n}\right)$, with the degree of the polynomial considered to represent $F_{j}^{i}\left(\mu_{n}\right)$ increasing with the separated representation mode *i*, i.e., the higher *i*, the higher the polynomial degree considered to represent $F_{j}^{i}\left(\mu_{n}\right)$ in order to avoid overfitting.

In order to calculate the approximation unknowns $w_{j}^{i,n}\left(x,t\right)$, we consider the extended collocation form, within the sPGD rationale

(5) $$\int_{\Omega_{\mu }}u^{*}\left(x,t;\mu \right)\sum_{k}\delta_{\mu_{k}}\left(\mu \right)\;\left(u\left(x,t;\mu \right)-\sum_{k}u_{k}\left(x,t\right)\delta_{\mu_{k}}\left(\mu \right)\right)d\mu =0,$$

with $\delta_{\mu_{k}}\left(\mu \right)$ the Dirac mass located at $\mu_{k}$.

## 2. Parametric Electromagnetic Fields

In the present context, the transmitter emits waves at a constant frequency in a direction denoted by an angle θ between $0^{\circ }$ and $90^{\circ }$. For each component of the electric and magnetic fields, the data generated by the simulations consists of an array of $N\times N_{\theta }$ values, *N* being the number of points in the spatial discretization and $N_{\theta }$ the number of points in the angular discretization. The aim of this work is to provide a method to determine through the values of the electromagnetic field in all *N* discretized points in the geometry for any value of θ between $0^{\circ }$ and $90^{\circ }$. In what follows, we will consider the problem of constructing the interpolation of the z-component of the electric field, $E_{z}$, for all θ in one point of the geometry (on a grid of points will be considered later).

### 2.1. Real-Imaginary Interpolation Versus Magnitude-Phase Interpolation

Since the solutions provided by the solver are in the frequency domain, they are complex-valued functions. Complex numbers can be represented either by their real and imaginary parts or by their magnitude and phase, therefore the interpolation of complex-valued functions can be computed in multiple ways which are not equivalent. The first option is to treat the real and imaginary parts as two real-valued functions, interpolate them separately and combine the results. The second option is to treat the magnitude and phase as two real-valued functions, interpolate them separately and combine the results.

**Figure 1:**
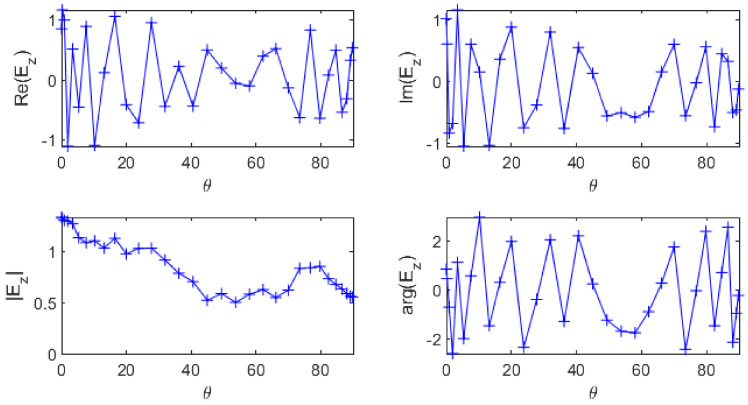
Real and imaginary parts (**top left** and **right** respectively), magnitude and phase of $E_{z}$ (**bottom left** and **right** respectively) as a function of θ in one point of the geometry.

The performances of these two methods depend on the considered problem. In our case, the data from the simulation have real and imaginary parts oscillating quite fast compared to the sampling frequency (Figure 1), hence they are not good candidates for interpolation. However the magnitude is much smoother which means it may give trustworthy interpolation results. The phase looks more chaotic but it is not a good indicator because of its natural 2π-periodicity that induced spurious discontinuities: the phase needs to be “unwrapped” before being interpolated.

### 2.2. Phase Unwrapping in Smooth Parametric Settings

Phase unwrapping is the reconstruction of a “physically-meaningful” representation of the phase (as a function of the parameter θ) by adding multiples of 2π to some of its values in order to make it a continuous function. This step is very important because it determines the number of periods between two successive values of θ, regardless of the interpolation method used. The unwrapped phase is defined as the unique representation of the phase which can lead to a correct continuous interpolation. Unwrapping has been extensively addressed, the interested reader can refer to [26,27] and the numerous references therein.

The goal is to find a sequence ${\left(k_{n}\right)}_{1\leq n\leq N_{\theta }}\in Z^{N_{\theta }}$, such that the unwrapped phase $\phi \in R^{N_{\theta }}$ verifies:

$$\forall n\in \left[1,N_{\theta }\right],\;\phi_{n}=\mathrm{Arg}E_{z}\left(\theta_{n}\right)+2k_{n}\pi$$

where Arg is the principal value of the phase in the interval (−π,π], under some regularity constraint.

The proposed solution consists of assuming the derivative of the phase does not vary too much, or, to put it another way, that the second derivative remains small. This hypothesis discussed later leads to a minimization of the variation of the derivative using a finite differences scheme to compute sequentially the values of the unwrapped phase:

(6) $$\begin{array}{cc} \; & k_{1}=0\\ \; & k_{2}=\underset{k\in Z}{\mathrm{argmin}}\;\left|\mathrm{Arg}\left(E_{z}\left(\theta_{2}\right)\right)+2k\pi -\phi_{1}\right|\\ \forall n\geq 3,\; & k_{n}=\underset{k\in Z}{\mathrm{argmin}}\;\left|\frac{\mathrm{Arg}\left(E_{z}\left(\theta_{n+1}\right)\right)+2k\pi -\phi_{n}}{\theta_{n+1}-\theta_{n}}-\frac{\phi_{n}-\phi_{n-1}}{\theta_{n}-\theta_{n-1}}\right| \end{array}$$

Note that $\theta_{2}$ must be chosen close enough to $\theta_{1}$ to ensure $|\phi_{2}-\phi_{1}|<\pi$. This algorithm can also be run in descending *n* order, for example to avoid initialization in a noisy area.

**Figure 2:**
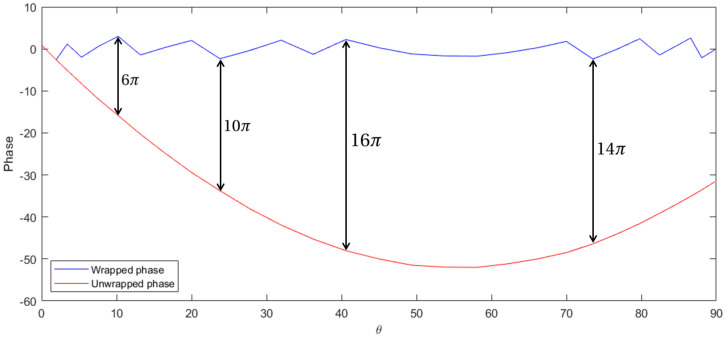
Example of successful phase unwrapping.

Once applied to our problem, phase unwrapping produces a very smooth curve, which allows great performance for interpolation (Figure 2).

The functioning interval for the just proposed strategy depends on the difference of the phase derivative between two consecutive sampling points, with respect to the sampling points distance. Thus, one can expect that $\Delta \phi^{\prime }\cdot \Delta \theta <C$, with C=π from our numerical experiments. In order to enlarge the applicability domain, an important enhancement is proposed in Section 2.

### 2.3. Robustness Issues

The phase unwrapping method discussed in the previous section performs well on smooth data but it lacks robustness, mainly for two reasons:

1. Even with very regular data, the phase can have a chaotic behavior when the magnitude is close to zero, which means our hypothesis on the regularity of the phase is not valid.
2. Since the different phase values are computed sequentially using the previous ones, local irregularities result in high global error.

However, because a failure of the method is caused by the invalidity of the hypothesis, computing the finite differences approximation of the second derivative of the phase is a good way to detect when the unwrapping fails.

### 2.4. Comparing the Proposed Algorithm with Traditional Unwrapping

**Figure 3:**
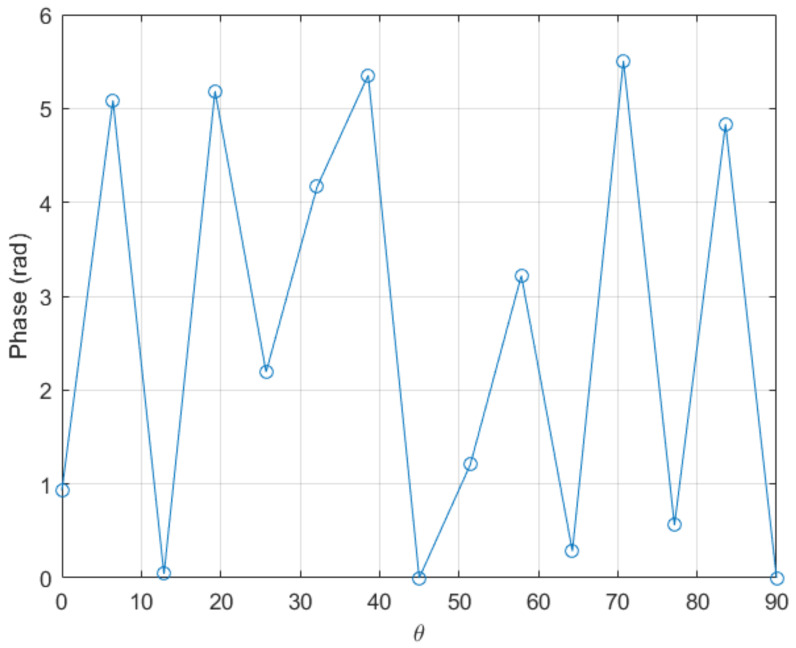
Original wrapped function.

To illustrate the advantages of the just introduced methodology, the function having as unwrapped form

(7) $$\phi =\left(\theta +10\right)\left(\theta -90\right)\left(\theta -45\right)/k_{f}$$

with $k_{f}=3000$, is considered. Figure 3 depicts the wrapped counterpart of the above function. For unwrapping it, we compare the proposed algorithm with the standard procedure included in the commercial software Matlab, the unwrap() function. The Matlab function applies when the difference between consecutive angles is greater than π, and shifts the angles by adding multiples of ±2π until the difference becomes less than π [30]. As noticed that function achieves a good unwrapping if a fine enough mesh is used (involving more than 35 nodes). However, the Matlab function fails to perform correctly in the case of coarser meshes (less than 30 nodes).

**Figure 4:**
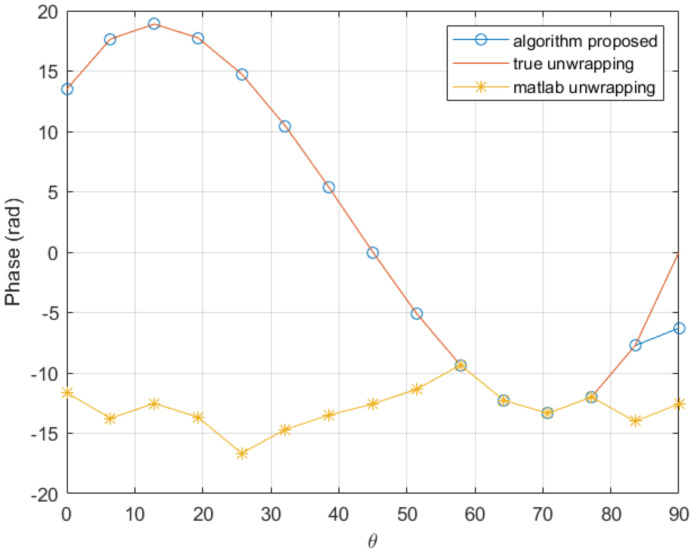
Comparison of the proposed unwrapping procedure and the standard unwrapping function implemented in Matlab, in a coarse mesh consisting of 15 nodes.

On the other hand, the procedure proposed in this paper achieves good results with only 15 nodes, as it can be seen in Figure 4, with the associated computing time saving.

**Figure 5:**
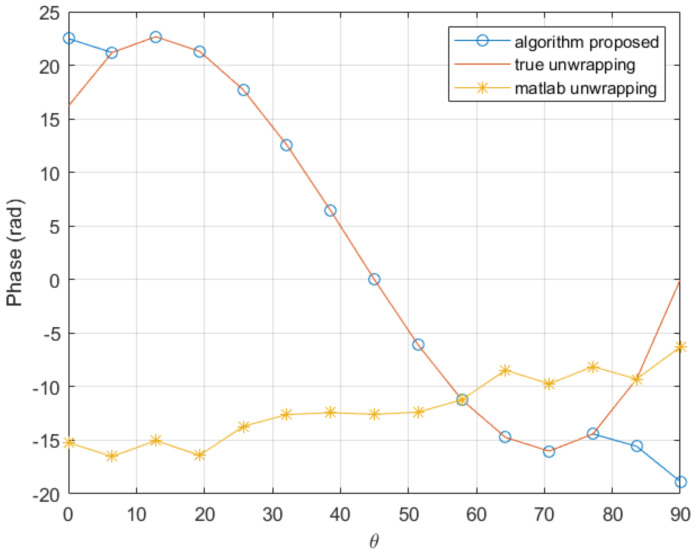
Comparison of the proposed procedure and the Matlab unwrapping function in a coarse mesh consisting of 15 nodes with the $1/k_{f}$ factor 20% higher.

**Figure 6:**
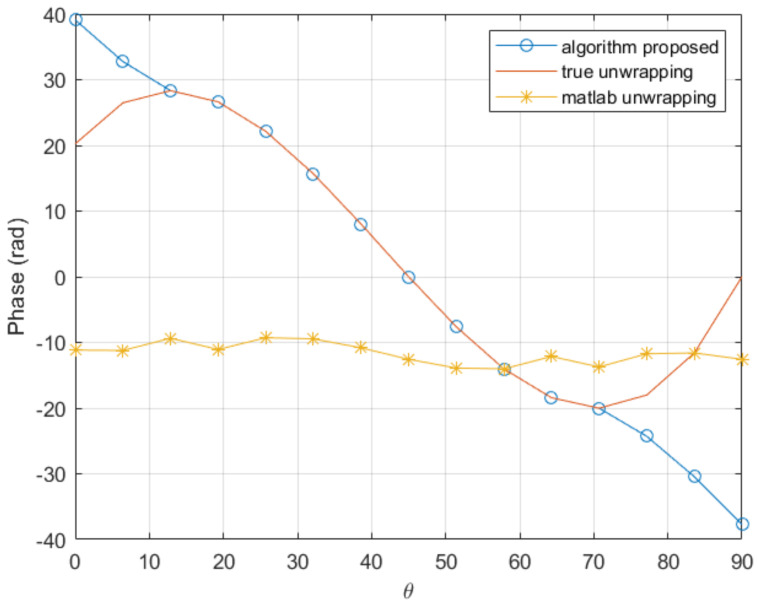
Comparison of the proposed procedure and the Matlab unwrapping function in a coarse mesh consisting of 15 nodes with the $1/k_{f}$ factor 50% higher.

In order to check the performances when the derivatives involved in the solution increase, we consider the previous function while increasing the factor $1/k_{f}$ of 20% and 50%, with the same coarse mesh consisting of 15 nodes. The comparison is shown in Figure 5 and Figure 6. As it can be noticed, the proposed procedure seems quite robust when compared with traditional unwrapping.

## 3. Improving the Robustness of Phase Unwrapping

So far, we have solved part of the original problem: phase unwrapping can be attempted on all *N* points in the spatial discretization and accepted or rejected by setting a threshold for the values of the second derivative. Thus, a part of the geometry has therefore been dealt with. In this section, we will discuss how to use these solved points to correct the phase unwrapping in the rest of the geometry.

### 3.1. Reduced Basis of the Search Space

The geometry can be divided into subdomains inside which the variations of the electromagnetic field are small. In each subdomain, the search space is reduced using the proper orthogonalized decomposition (POD) computed with the method of the snapshots where the snapshots are the unwrapped phases from the points in the subdomain which are already solved. The local coherence of the electromagnetic field allows the POD to have a very low dimension, typically 2 or 3, denoted in the following as *r*.

### 3.2. Phase Unwrapping in the Reduced Search Space

Out of all the possible configurations of the phase, we are looking for the one closest to the reduced search space. This can be expressed as a minimization problem:

(8) $$\min_{\underset{\alpha \in R^{r}}{k\in Z^{N_{\theta }}}}∥\mathrm{Arg}\left(E_{z}\right)+2\pi k-\sum_{i=1}^{r}\alpha_{i}w_{i}∥$$

where ${\left(w_{i}\right)}_{i=1,\ldots ,r}$ are the basis vectors of the search space and ${\left(\alpha_{i}\right)}_{i=1,\ldots ,r}$ are the vector coefficients with respect to this base.

The search of *k* can easily be limited to ${\left[k_{-},k_{+}\right]}^{N_{\theta }}\subset Z^{N_{\theta }}$, $k_{-}$ and $k_{+}$ being two integers which depend on the size of the unwrapping. However, this problem can still be very hard to solve hence we will approximate the resolution.

The proposed algorithm to work around this high complexity follows three main steps:

1. Using only a few of the raw phase values, generate a discrete subset of the search space which is likely to be close to the optimal solution.
2. Fit the rest of the phase values to each curve of this subset by adding or subtracting multiples of 2π.
3. Select the configuration which allowed for the best fit.

Thus, *r* points ($\theta_{n_{1}},\ldots ,\theta_{n_{r}}$) are chosen from the θ discretization. For $\left(k_{n_{1}},\ldots ,k_{n_{r}}\right)\in {\left[k_{-},k_{+}\right]}^{r}$, we can find the unique curve in the search space which can be fit to the *r* points $\left(\mathrm{Arg}\left(E_{n_{1}}\right)+2\pi k_{n_{1}},\ldots ,\mathrm{Arg}\left(E_{n_{r}}\right)+2\pi k_{n_{r}}\right)$, by solving for the coefficients $\alpha_{i}$:

(9) $$\forall j\in ⟦1,r⟧,\;\sum_{i=1}^{r}\alpha_{i}w_{i}\left(\theta_{n_{j}}\right)=\mathrm{Arg}\left(E_{n_{j}}\right)+2\pi k_{n_{j}}$$

In practice, we choose the $n_{r}$-tuple ($\theta_{n_{1}},\ldots ,\theta_{n_{r}}$) minimizing the condition number of the linear system represented by Equation (9). Let $\alpha_{i}:\left(k_{n_{1}},\ldots ,k_{n_{r}}\right)\mapsto \alpha_{i}\left(k_{n_{1}},\ldots ,k_{n_{r}}\right)$ be the function that associates with each configuration the vector coefficients solution to the linear system (9). We can now solve instead of (8) the following problem:

(10) $$\min_{\left(k_{n_{1}},\ldots ,k_{n_{r}}\right)\in {\left[k_{-},k_{+}\right]}^{r}}\;\min_{k\in Z^{N_{\theta }}}∥\mathrm{Arg}\left(E_{z}\right)+2\pi k-\sum_{i=1}^{r}\alpha_{i}\left(k_{n_{1}},\ldots ,k_{n_{r}}\right)w_{i}∥$$

### 3.3. Validation of the Optimization Procedure

We consider the following phase function

ϕ(θ)=8πsin(4θ)−4.5πsin(8θ)+sin(12θ),

involving 30 points in the discretization of the θ parametric coordinate.

Once it is artificially wrapped, both the standard unwrapping algorithm and the procedure proposed in this paper based on the second derivative, fail to unwrap it.

**Figure 7:**
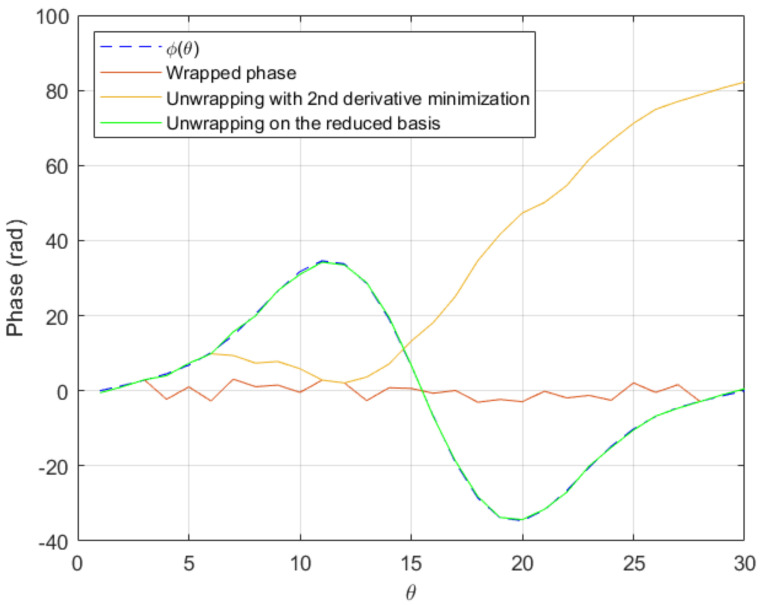
Comparison of reduced-basis based unwrapping versus second derivative minimization based unwrapping on a test case exhibiting fast variations of the phase.

Assuming we have computed the reduced basis consisting of the three functions {sin(4θ),sin(8θ),sin(12θ)}, we attempt to unwrap $\mathrm{Arg}\left(e^{i\phi (\theta )+N(0,0.5)}\right)$ using the just described optimization procedure. The unwrapped phase is exactly the sum of ϕ(θ) and the added noise, as Figure 7 proves.

## 4. Numerical Results

### 4.1. Convergence Analysis

Since the simulations of radar devices are too expensive, in this section we consider a simpler problem able to provide the required data for performing the convergence analysis.

Thus, we consider the Helmholtz problem with damping in a 2D rectangular domain Ω, that looks for u(x,y) verifying

(11) $$\left\{\begin{array}{cc} \Delta u+(i\nu \omega -\omega^{2})u=f & \;\mathrm{in}\;\Omega \\ \frac{\partial u}{\partial n}=0 & \;\mathrm{on}\;\partial \Omega \end{array}\right.$$

where Ω=[0,9]×[0,2], ω=10, ν=1 and $f\left(x,y;\theta \right)=e^{120i(cos(\theta )x+sin(\theta )y)}$.

**Figure 8:**
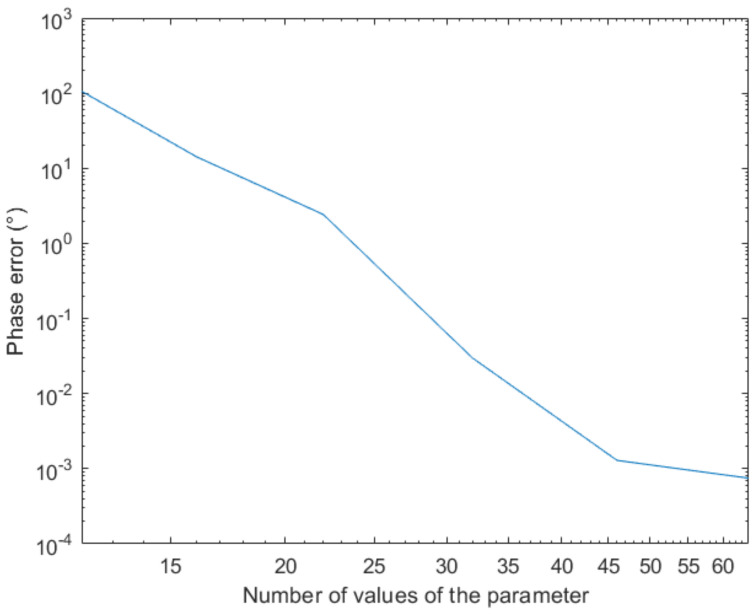
Evolution of the phase error with the parameter sampling refinement.

We discretize and solve this problem using the Finite Element Method (FEM) with Lagrange P1 elements for a number of values of θ, and interpolate the solution (complex field) at $\theta =3^{\circ }$. Then, we compare the phase of the interpolated field to the phase of the solution obtained with the FEM, whose difference is depicted in Figure 8.

The previous figure proves the convergence of the proposed strategy when enriching the sampling. It can also be noticed that a quite sparse sampling suffices for reaching an acceptable accuracy.

### 4.2. Results on the RADAR Problem

**Figure 9:**
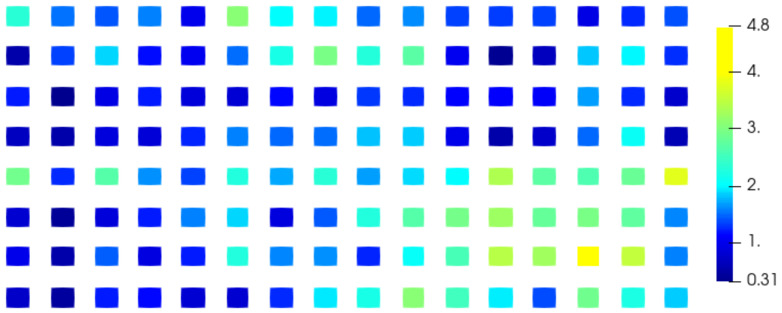
Mean absolute error on the phase (${}^{\circ }$) of the $E_{z}$ component over each patch of the antenna using the proposed method.

**Figure 10:**
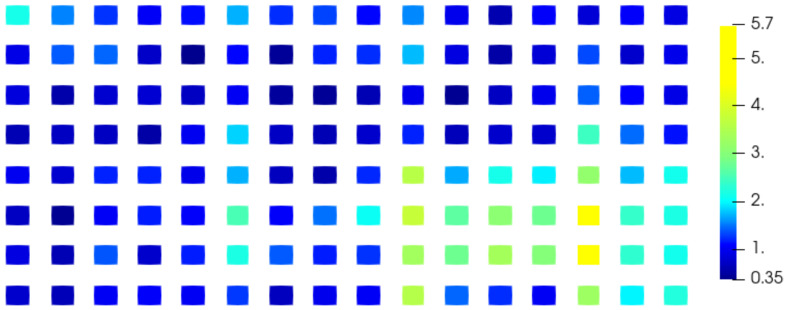
Mean absolute error on the phase (${}^{\circ }$) of the $H_{y}$ component over each patch of the antenna using the proposed method.

**Figure 11:**
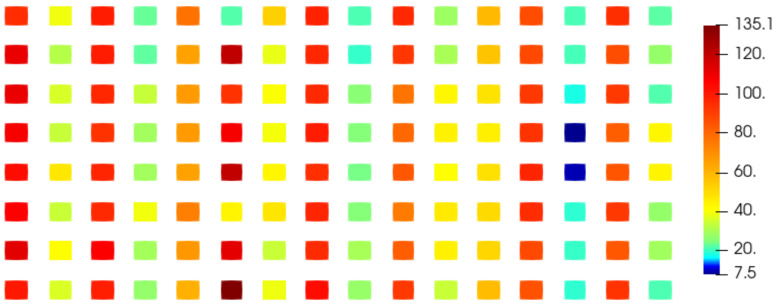
Mean absolute error on the phase (${}^{\circ }$) of the $E_{z}$ component over each patch of the antenna using the classical unwrapping algorithm.

**Figure 12:**
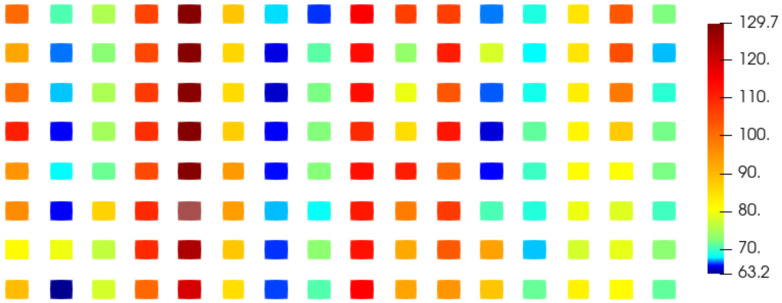
Mean absolute error on the phase (${}^{\circ }$) of the $H_{y}$ component over each patch of the antenna using the classical unwrapping algorithm.

The electromagnetic field on a patch antenna (8×16 rectangular patches) has been computed for 33 different values of θ between $0^{\circ }$ and $90^{\circ }$, 32 of which are used to apply our method and one as reference to compare the interpolation with the simulation. The reference electromagnetic field corresponds to the parameter value $\theta_{ref}=79.8^{\circ }$. The two closest values of theta used to compute the interpolation are $76.8^{\circ }$ and $82.4^{\circ }$ hence $\theta_{ref}$ lies in a gap of size $\Delta \theta =82.4^{\circ }-76.8^{\circ }=5.6^{\circ }$. The values of the electromagnetic field below 0.2 V/m for the electric component (E) and 0.5 mA/m for the magnetic component (H) are not considered in this study because the signal is not considered significant enough and the phase cannot be studied because of its singularity (Appendix A). For this reason and due to the polarization of the electromagnetic field, only the z-component of the electric field $E_{z}$ and y-component of the magnetic field $H_{y}$ provide results. We calculate the absolute error between the phase of the reference field and the phase of the interpolated field and plot the mean error over each patch of the antenna using our method (Figure 9 and Figure 10) and the classical unwrapping algorithm for comparison (Figure 11 and Figure 12).

With the proposed method, the average error over the entire antenna is $1.6^{\circ }$ for the electric field and $1.4^{\circ }$ for the magnetic field while with the standard unwrapping algorithm, the average error over the entire antenna is $62.0^{\circ }$ for the electric field and $63.7^{\circ }$ for the magnetic field. In comparison, the average error on the phase when interpolating real and imaginary parts is $166.8^{\circ }$ for the electric field and $166.7^{\circ }$ for the magnetic field.

Our method is accurate and robust as the mean phase error is consistently under $6^{\circ }$ on the entire antenna for both the electric and magnetic fields, whereas real and imaginary parts interpolation completely fails when dealing with such a sparse discretization of the parameter θ.

## 5. Conclusions

In this paper, we tackled the computation of parametric solutions for electromagnetic wave propagation in radar applications. These simulations currently require hundreds of hours of computations to obtain suitable accuracy.

We have described an interpolation method for complex-valued fields which allows reducing the computational cost significantly. By exploiting the natural regularity of electromagnetic fields, we are able to retrieve highly accurate parametric solutions from a very sparse set of simulations.

## Figures and Tables

**Figure A1:**
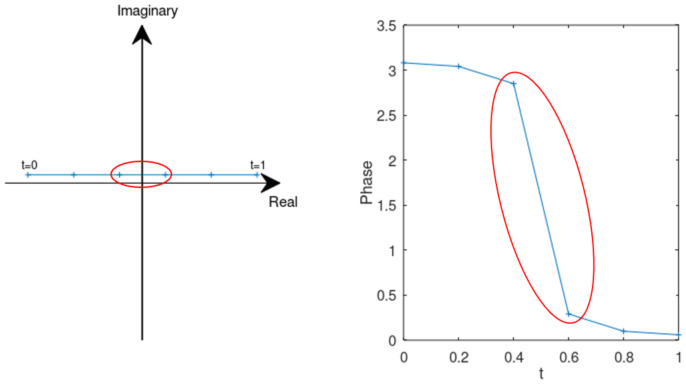
Example of a smooth parametric curve on the complex plane (**left**) and the phase as a function of the parameter (**right**).

**Figure A2:**
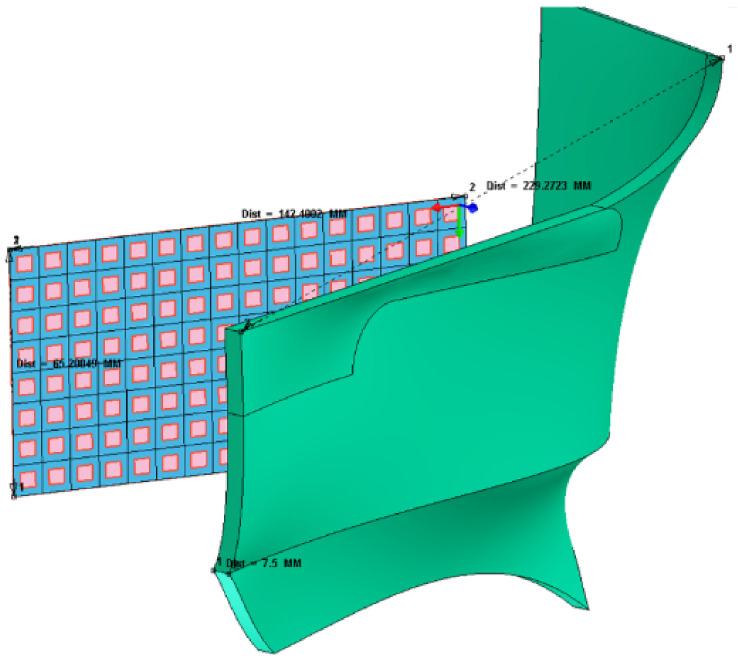
Radar antenna and radome.

## Appendix A. Phase Singularity

The phase of a complex-valued function can have very fast variations when its magnitude is close to zero, even if the function is very regular (Figure A1). This may induce large error in the interpolation of the phase, which cannot be easily avoided. However since the magnitude is very small, a large error on the phase does not have too much impact on the complex number itself, which can usually simply be regarded as zero.

## Appendix B. Antena Modelling and Simulation

The simulation model discussed in this paper is illustrated in Figure A2. This simplified mock-up represents the receiving part of a typical automotive radar device operating at 24 GHz and located behind a plastic bumper (Blind Spot Detection).

The antenna is made of 16×8 square array elements printed on a rectangular substrate (thickness about 0.254 mm with a dielectric permittivity set equal to 2.2 and an electrical conductivity of 0.003 S/m). The average thickness of the plastic bumper is 7.5 mm and its permittivity about 2.6 (its conductivity being negligible).

As mentioned in the paper, computational results were obtained using the CEM-TD product included in the CEM One^®^ software package and based on the standard FDTD simulation technique (Finite Difference Time Domain).

Once the device is illuminated by an external plane wave, electric and magnetic field components can be thus accessed at the level of each basic antenna element and thus expressed in frequency domain (modulus and phase) using a classical Fourier transform—see for instance Figure 9 and Figure 10.

With an incidence angle of the 24 GHz exciting wave ranging from $0^{\circ }$ to $90^{\circ }$ in the horizontal plane (illustrated in Figure A2 for a given sample of the array antenna), the spureous effect of the plastic bumper can be evaluated by comparing the fields actually received by the antenna with the reference signal obtained without any extra obstacle.
